# Crop yield and water use efficiency in response to long-term diversified crop rotations

**DOI:** 10.3389/fpls.2022.1024898

**Published:** 2022-10-07

**Authors:** Zhengjun Cui, Bin Yan, Yuhong Gao, Bing Wu, Yifan Wang, Yaping Xie, Peng Xu, Haidi Wang, Ming Wen, Yingze Wang, Xingkang Ma

**Affiliations:** ^1^ State Key Laboratory of Arid Land Crop Science, Lanzhou, China; ^2^ College of Agronomy, Gansu Agricultural University, Lanzhou, China; ^3^ College of Life Science and Technology, Gansu Agricultural University, Lanzhou, China; ^4^ Crop Research Institute, Gansu Academy of Agricultural Sciences, Lanzhou, China

**Keywords:** diversified crop rotations, soil water, evapotranspiration, crop yield, water use efficiency

## Abstract

Crop production and water productivity may be impacted by diverse crop rotation and management practices. A field study was conducted from 2017-2020 in the Loess Plateau to evaluate the effects of crop rotation sequences on pre-planting and post-harvest soil water storage (SWS), annualized crop yield, water use, and water productivity. Crops in rotation included oil flax (*Linum usitatissimum* L.) (F), wheat (*Triticum aestivum* L.) (W), potato (*Solanum tuberosum* L.) (P). Twelve 4-year-cycle crop rotation treatments, along with a continuous oil flax treatment as a baseline, were included. The results showed that the average soil water content under the 0-150 cm soil layer in all treatments was increased after one rotation cycle, and the PWFW treatment achieved the highest SWC (17.1%). The average soil water storage (winter fallow season) and evapotranspiration (ETa) (growing season) under different crop rotation sequences were lower than those under continuous oil flax cropping. The ETa of FFFF increased by 28.9, 2.7, 15.3, and 28.4%, compared to average crop rotations in 2017, 2018, 2019, and 2020, respectively. Crop rotation had a significant effect on average annual yield and water use efficiency (WUE), which varied by year and rotation sequence. The crop rotations with the highest grain yield of oil flax were FFWP (2017), WFWP (2018),WPFF (2019) and FWPF (2020); the grain yield of wheat was highest when the two pre-crops (previously cultivated crops) were F-F, and potato yield was highest when the two pre-crops were W-F (except 2018). On average, the WUE of oil flax was 8.6, 38.7, 22.7, and 42.1% lower with FFFF than other diversity crop rotations in 2017, 2018, 2019, and 2020. We found that the WUE was not the largest when the grain yield of oil flax and wheat was highest. The treatments with maximum grain yield and WUE were not consistent. Our findings also revealed that wheat-potato-oil flax or potato-wheat-oil flax rotation could increase oil flax grain yields while wheat-oil flax-potato-oil flax markedly improved oil flax WUE.

## Introduction

Flax (*Linum usitatissimum* L.) is an ancient crop grown for seed and oil and for the strong fiber produced in its stems (Ehrensing, 2008). It is cultivated in many countries worldwide (Zuk et al., 2015). With growing interest in oil flax for use in functional foods, dietary supplements, and biodiesel production, there is an increasing need to identify strategies to enhance its productivity to meet the growing demand (Xie et al., 2020a). Apart from its potential functional value, oil flax has drought tolerance. It grows well in permeable soils with good water holding capacity and a period of cold temperatures  (Ehrensing, 2008). Nevertheless, its cultivation is also challenged by drought and insufficient nutrient availability in the semiarid soil owing to high evaporation and erratic rainfall that cause under-or over-water supply. Another problem oil flax production faced was the continuous cropping obstacle. The grain yield of oil flax decreased significantly after 2-3 years of continuous cropping (Wang et al., 2022). In addition, continuous cropping of oil flax reduced seed germination, inhibited plant growth, and adversely affected some soil enzyme activities, and continuous cropping years were significantly negatively correlated with soil nutrients and enzyme activities (Wang et al., 2021).

Sustainable crop production in arid and semi-arid regions of the world is severely limited by inadequate precipitation, short growing seasons, and highly variable climatic conditions and single planting patterns (Liebig et al., 2015). For example, in the North Eastern Region of India, the Loess Plateau of China, the Great Plains of the US, alternate crop-fallow rotation and continuous mono-cropping are conventional cropping systems still practiced by many producers (Potter et al., 1997; Das et al., 2018; Peng et al., 2020; Xie et al., 2020b; Li et al., 2021a). However, continuous cropping obstacles often appear in continuous cropping patterns after a few years of use, even with a good field management regime (Tan et al., 2021). Long-term continuous planting will bring a series of problems to the soil, especially the partial consumption of soil nutrients and the decline of water use efficiency (Tan et al., 2021). Crop production primarily depends on fertility and water of soil, and sustained crop productivity relies on constant renewal of water supply (Wang & Shangguan, 2015), crops yield is largely determined by water. There is research that shows that crops yields have been negatively impacted by water scarcity in the past 30 years (Li et al., 2009). The water shortage issue is expected to worsen in the future (Liu et al., 2016).

Crop rotations have been repeatedly shown to be effective methods of improving water use efficiency and maintaining sustainable crop production (Sun et al., 2018). This may be because crop rotation enables optimization of nutrient use, reduction of pest and disease infections, and increases crop yields by efficiently using soil water (Dias et al., 2015). Studies have shown that SWS, crop yield, WUE, and water productivity were greater with different crop rotation sequences than with mono-cropping (Schlegel et al., 2017; Lenssen et al., 2018). In addition, crop rotation not only decelerated soil nutrient depletion, but also maintained dry-land crop yields and fully utilized precipitation (Sainju et al., 2021). It is not enough to evaluate the effectiveness of dry-land cropping systems based solely on crop yield and profitability, and water productivity should be included in the assessment of crop rotation benefits (Schlegel et al., 2019). This may be because crop yields and profit are often affected by climatic conditions, total input and output, and price fluctuations in addition to diversified crop rotation systems (Toliver et al., 2012; Schlegel et al., 2016). Therefore, diversified crop rotation systems in dry-farming region are more sustainable if supported by improved water productivity rather than solely on crop yields and profits (Schlegel et al., 2019).

Annual spring maize (*Zea mays* L.), wheat, potato, oil flax, and winter fallow are the prevailing crop systems in the Loess Plateau due to climate and geographical conditions, while neither oil flax nor potato can be grown in continuous cropping (Liu et al., 2016; Zhao et al., 2020a). Therefore, it is particularly important to have a well-designed and successful crop rotation to increase crop yield and water productivity, improve the soil environment, decrease agricultural inputs, and enhance the profit of the producer. At the same time, a long and diverse crop rotation helps alleviate the self-intolerance of crops due to continuous cropping (Zhao et al., 2020a). Unlike other studies, such as evaluation of a certain fertilizer amount, planting density or irrigation amount, crop rotation studies require data for a long time because of the need to complete one or more cycles of multi-year rotations (Schlegel et al., 2018). Due to the possible variations in the farm microclimate short-term crop rotation studies may obtain inaccurate results (Nielsen and Vigil, 2014).

Because of the reduction in crop yields (Álvaro-Fuentes et al., 2009) and low water use efficiency (Pala et al., 2007) due to conventional continuous mono-cropping, improved management strategies are needed to enhance SWS, crop yields, evapotranspiration, and WUE in the semiarid regions. It is demonstrated that crop rotation with wheat can serve as an effective means to reduce the continuous cropping obstacle of oil flax (Wang et al., 2020). Only one crop of oil flax was grown in 4 growing seasons could maintain the stability of soil organic carbon, which was identified as the ideal rotation sequence for dry-land oil flax crops (Liu et al., 2018). However, few studies have been performed to examine the impact of diversifying crop rotations on the grain yield and water use efficiency of oil flax. Therefore, the aim of this study was to evaluated the effect of diversified crop rotation sequences on pre-planting and post-harvest soil water content, fallow soil water storage, annualized crop yield, water productivity, and relative coefficient variation from 2017 to 2020 in the semiarid Loess Plateau, China. We hypothesized that the diversified crop rotations would increase grain yield and WUE of oil flax. The specific objectives were to: (1) investigate the effects of crop rotation on SWS, crop yield, water use, and water productivity; and (2) identify the best crop rotation sequences. In contrast to continuous mono-cropping, we expected that diverse crop rotation and the frequency of oil flax would boost crop yield, water use, and water productivity.

## Materials and methods

### Study site description

**Figure 1:**
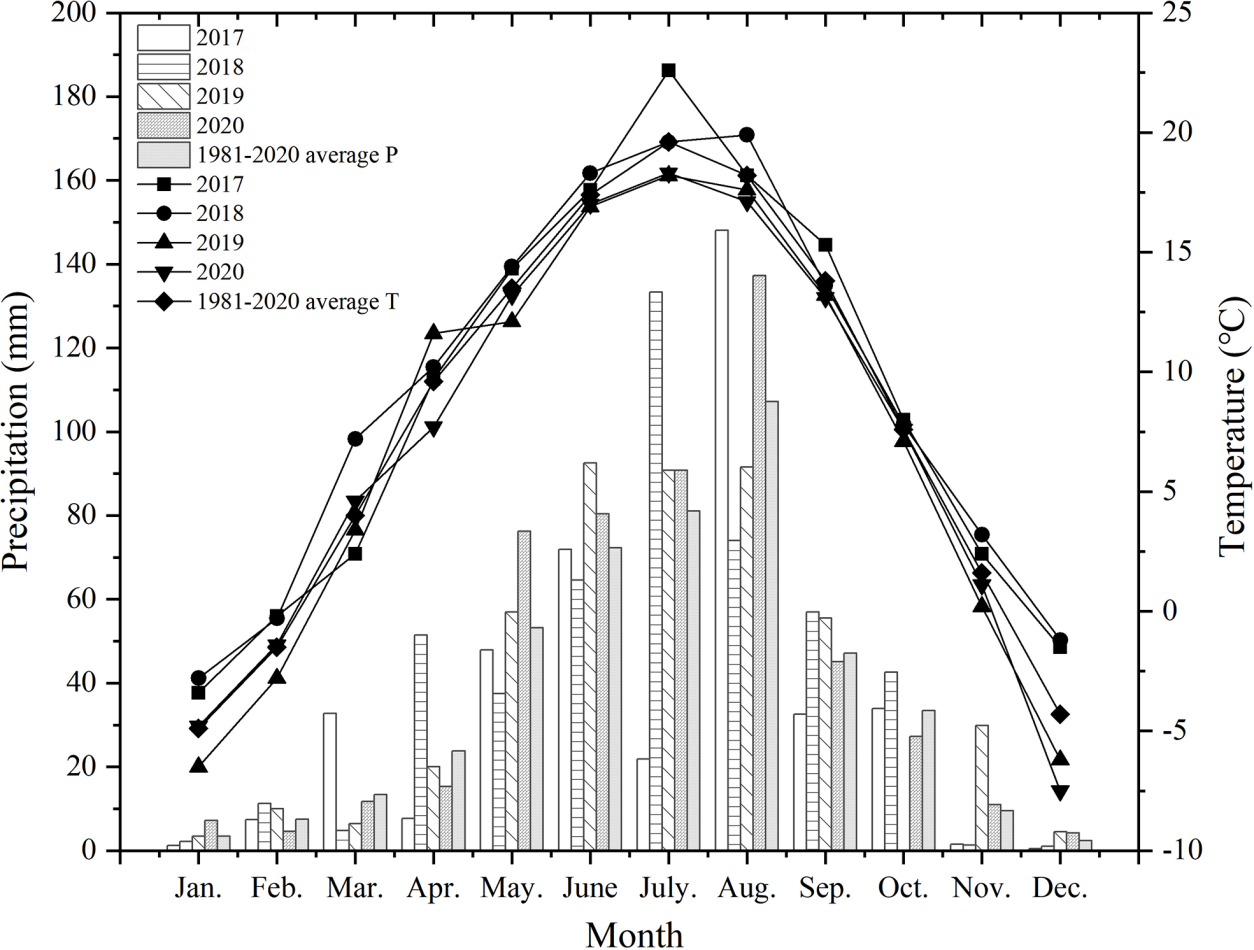
Average monthly precipitation and temperature from 2017 to 2020. P, Precipitation; T, Temperature.

Using data from a long-term field experiment that was initiated in 2013, the current investigation focused on the 2017 to 2020 growing seasons. The field research was carried out at the Dingxi Academy of Agricultural Science in Gansu Province’s Anding District, which is located at 103° 52′ E and 34° 26′ N. The annual mean temperature is 6.3 degrees Celsius, and there are typically 2453 hours of sunshine per year. The average yearly precipitation during the past 30 years was 377 mm (the data was derived from the Dingxi Weather Station).The annual precipitation displays an uneven characteristic, with nearly 60% occurring during June-September. During the study years (2017-2020), the mean annual temperature at the site was 8.04 °C and the mean annual precipitation was 465.3 mm. The climate details for this experiment are shown in **Figure 1**.

Prior to the start of this experiment, the site was planted in oil flax, and conventional tillage was applied every year after crop harvest. Soil samples were collected randomly at five points to determine the contents of total nitrogen (N), total phosphorus, soil organic matter, Alkali-hydrolyzable N, available phosphorus, available potassium, and pH of the 0-30 cm soil layer: 1.00, 0.81, and 17.51g kg^-1^ and 47.91, 26.43, and 108.30 mg kg^-1^ and 8.13, respectively.

### Experimental design and treatments

From 2017 to 2020, one cycle of the four-year crop sequences was carried out (**Table 1**). The basic treatment of the design was Phase1 using the internationally accepted crop rotation experiment design method, and then generating Phase2, Phase3, and Phase4 based on Phase1. Each phase was divided into four crop rotation sequences according to the frequency occupied by oil flax: (1) 100% oil flax, (2) 50% oil flax (I), (3) 50% oil flax(II), and (4) 25% oil flax. The types and numbers of crops in the 4 phases were the same, but the order of crops was different. For example, the 50% oil flax(I) rotation sequence in Phase I was FWPF, which was adjusted to WPFF in Phase II, the first crop in Phase I was adjusted to the last crop in Phase II, and so on, adjusted to PFFW in Phase III and FFWP in Phase IV, a total of 16 cropping systems were obtained, and 4 were continuous oil flax (**Table 1**). All treatments were arranged in a randomized complete design with three replications. The area of each plot was 3 m x 5 m = 15 m^2^. **Tables 2** and **3** describe the planting and harvesting details of the 4-year rotations.

**Table 1:** The 4-yr cropping sequences for one cycles.

Phase	Crop rotation	Crop year				Oil flax frequency
		2017	2018	2019	2020	
Phase I	FFFF	Oil flax	Oil flax	Oil flax	Oil flax	100%
	FWPF	Oil flax	Wheat	Potato	Oil flax	50%(I)
	FPFW	Oil flax	Potato	Oil flax	Wheat	50%(II)
	FWPW	Oil flax	Wheat	Potato	Wheat	25%
Phase II	FFFF	Oil flax	Oil flax	Oil flax	Oil flax	100%
	WPFF	Wheat	Potato	Oil flax	Oil flax	50%(I)
	PFWF	Potato	Oil flax	Wheat	Oil flax	50%(II)
	WPWF	Wheat	Potato	Wheat	Oil flax	25%
Phase III	FFFF	Oil flax	Oil flax	Oil flax	Oil flax	100%
	PFFW	Potato	Oil flax	Oil flax	Wheat	50%(I)
	FWFP	Oil flax	Wheat	Oil flax	Potato	50%(II)
	PWFW	Potato	Wheat	Oil flax	Wheat	25%
Phase IV	FFFF	Oil flax	Oil flax	Oil flax	Oil flax	100%
	FFWP	Oil flax	Oil flax	Wheat	Potato	50%(I)
	WFPF	Wheat	Oil flax	Potato	Flax	50%(II)
	WFWP	Wheat	Oil flax	Wheat	Potato	25%

**Table 2:** Planting and harvesting information of oil flax, wheat, potato in the rotation systems.

Crop year	Crops	Variety	N-P_2_O_5_ Fertilizer (kg ha^-1^)	Seeding rate(per m^2^)	Planting date	Harvest date	Growth period (d)	Precipitation (mm)
2017	oil flax	Longya 10	112.5-75	750	16-Apr	21-Aug	127	224.0
	Wheat	Ganchun 27	150-112.5	375	22-Mar	1-Aug	132	155.0
	Potato	Xindaping	225-150	5.25	4-May	4-Oct	153	322.4
2018	oil flax	Longya 10	112.5-75	750	7-Apr	20-Aug	135	352.9
	Wheat	Ganchun 27	150-112.5	375	20-Mar	28-Jul	130	287.2
	Potato	Xindaping	225-150	5.25	7-May	29-Sep	144	478.4
2019	oil flax	Longya 10	112.5-75	750	8-Apr	19-Aug	133	303.4
	Wheat	Ganchun 27	150-112.5	375	24-Mar	30-Jul	128	169.5
	Potato	Xindaping	225-150	5.25	4-May	3-Oct	151	349.1
2020	oil flax	Longya 10	112.5-75	750	9-Apr	23-Aug	136	319.5
	Wheat	Ganchun 27	150-112.5	375	26-Mar	8-Aug	135	305.0
	Potato	Xindaping	225-150	5.25	14-May	27-Sep	135	401.1

Superphosphate (16%) and urea (46%) were used as the fertilizers for P_2_O_5_ and N, respectively. The fertilizers were administered once a year and evenly distributed across the soil surface before planting. The fertilizer application amounts are detailed in **Table 2**. For potato, wheat, and oil flax, the row spacings were 0.2 m, 0.2 m, and 0.3 m, respectively.

### Measurements

Soil water storage was calculated for a 1.5 m profile by multiplying the mean soil water content by the soil profile depth. The soil water content (SWC) was measured at 0-10, 10-30, 30-60, 60-90, and 90-150 cm intervals in the 0-150 cm soil profile for oil flax, wheat, and potato with soil cores randomly collected in each plot at the sowing and harvesting stages every year. The soil samples were collected by drilling equipment and loaded into aluminum boxes. The fresh weight was determined. The sample was oven-dried at 105°C for 24 h and then the dry weight was measured. The requisite indices were calculated according to the following formulae (Zhang et al., 2019a; Zhang et al., 2020).


(1) SWC (%) = (FW−DW) / (DW−AW)

where FW is the fresh weight of the soil sample with the aluminum box, DW is the dry weight of the soil sample with the aluminum box, and AW is the weight of the aluminum box.


(2) $$SWS\;\left(mm\right)\;=\;\sum SWC_{i}\cdot D_{i}\cdot H_{i}$$

where SWC (%) is the soil water content; D_i_ (g cm^-3^) is the soil bulk density; H_i_ (cm) is the soil depth. The average SWS from each sampling date was used in statistical analysis.


(3) $$ETa=\;SWS_{bf}-\;SWS_{h}+\;P_{i}$$

where SWS_bf_ is soil water storage before the sowing stage, SWS_h_ is soil water storage after harvesting and P_i_ is precipitation during the crop growing period. Evapotranspiration (ETa mm) is the water consumption during the crop-growing season.


(4) WUE = Y/ETa

where WUE (kg ha^-1^ mm^-1^) is the water use efficiency in the field; and Y (kg ha^-1^) is the economic yield. The crop yield was determined by manual harvesting, threshing, and air-drying grain from three 3 m^2^, 3 m^2^ and 7.5 m^2^ areas selected at random in each plot for oil flax, wheat and potato, respectively.


(5) $$\begin{array}{cc} RCV= & \sqrt{\frac{\sum_{\text{i}}^{n}\left(\text{Xi}-\text{X}\right)}{\text{n}-1}} \end{array}$$

The relative coefficient variation (RCV) was used to estimate the inter-annual fluctuation of grain yield or WUE, and measure crop production stability annually. Where the Xi is the annual relative grain yield or WUE, X is the average of relative yield or WUE, n is the experimental years, it is 4 in this case.


(6) $$\begin{array}{cc} X= & \frac{\text{yi}}{\text{Y}} \end{array}$$

where X are relative grain yield or WUE, yi is the grain yield or WUE in each experimental year, Y is the average grain yield or WUE in 4 experimental years.

### Statistical analysis

Three replications were calculated for each measurement, and ANOVA was used to compare the effects of different treatments on the measured variables. F-tests was conducted, and multiple comparisons were performed using the least significant difference test (LSD), (*P* ≤ 0.05). The experimental data were analyzed with the SPSS statistical package v.24.0 (SPSS Inst., Cary, NC), and the figures were generated using Origin 2019b (Systat Software Inc.).

## Results

### Crop yield

Oil flax, wheat, and potato yield were significantly affected by crop rotation sequences (*P*< 0.001), year (*P*< 0.001) and the interaction effects of year with crop rotation sequences (*P*< 0.001) during the growth seasons (**Table 3**).

**Table 3:** Effect of diversification crop rotation sequences on economic yield of oil flax, wheat and potato during 2017-2020.

Year	Flax		Wheat		Potato	
	Crop rotation	Yield (kg ha^-1^)	Crop rotation	Yield (kg ha^-1^)	Crop rotation	Yield (kg ha^-1^)
2017	FFFF	831.80c	WFPF	3134.01a	PWFW	36618.30b
2017	FWPF	859.07c	WPWF	2836.31b	PFFW	29314.65c
2017	FPFW	700.35d	WPFF	3149.91a	PFWF	42334.49a
2017	FFWP	1067.53a	WFWP	2955.92b		
2017	FWFP	932.47b				
2017	FWPW	979.16b				
2018	FFFF	650.63d	FWPF	2022.01b	WPWF	28214.10a
2018	WFPF	1335.67a	PWFW	1816.91c	WPFF	26835.63a
2018	PFFW	1140.57b	FWFP	1236.62d	FPFW	28847.75a
2018	PFWF	1210.61b	FWPW	2250.46a		
2018	WFWP	1398.37a				
2018	FFWP	825.75c				
2019	FFFF	1513.89d	WPWF	3606.67a	FWPF	34067.78b
2019	WPFF	2283.33a	PFWF	3811.11a	WFPF	34026.67b
2019	FPFW	2024.44b	WFWP	3780.00a	FWPW	37483.33a
2019	PWFW	2240.00a	FFWP	4126.67a		
2019	PFFW	1804.44c				
2019	FWFP	2162.22ab				
2020	FFFF	1207.50d	FPFW	2323.33a	WFWP	25451.11a
2020	FWPF	2157.78a	PWFW	1830.00b	FFWP	25222.22a
2020	WFPF	1957.78b	PFFW	2333.33a	FWFP	25584.44a
2020	WPWF	1944.44b	FWPW	1826.67b		
2020	WPFF	1396.67c				
2020	PFWF	1906.67b				
Average						
2017		895.06b		3019.04b		36089.15a
2018		1093.60b		1831.50c		27965.83b
2019		2004.72a		3831.11a		35192.59a
2020		1761.81a		2078.33c		25419.26b
ANOVA						
Variation		P-value				
Y		0.000(***)		0.000(***)		0.000(***)
CR		0.000(***)		0.000(***)		0.000(***)
Y × CR		0.000(***)		0.000(***)		0.000(***)

Numbers followed by different letters within a column in a set are significantly different at P = 0.05 by the LSD test. *** Significant at P ≤ 0.001, NS no significant difference.

FFFF, continuous oil flax; FWPF, oil flax-wheat-potato-oil flax; FPFW, oil flax-potato-oil flax-wheat; FFWP, oil flax-oil flax-wheat-potato; FWFP, oil flax-wheat-oil flax-potato; FWPW, oil flax-wheat-potato-wheat; WFPF, wheat-oil flax-potato-oil flax; PFFW, potato-oil flax-oil flax-wheat; PFWF, potato-oil flax-wheat-oil flax; WFWP, wheat-oil flax-wheat-potato; WPFF, wheat-potato-oil flax-oil flax; PWFW, potato-wheat-oil flax-wheat; WPWF, wheat-potato-wheat-oil flax. Y, year; CR, crop rotation.

### Oil flax

The grain yield improvements of 1110, 911 kg ha^-1^ and 867, 668 kg ha^-1^ in 2019 and 2020, compared with 2017 and 2018, respectively are remarkable. The variations in crop rotation patterns were noticeable throughout four growing seasons. The highest average oil flax yields were recorded in 2017, 2018, 2019, and 2020, respectively, by FFWP (1067.53 kg ha^-1^), WFWP (1398.37 kg ha^-1^), WPFF (2283.33 kg ha^-1^) and FWPF (2157.78 kg ha^-1^). FFFF (1050.96 kg ha^-1^) had the lowest average oil flax yield in four years (**Table 3**). The highest grain yield of oil flax was achieved in 2019 with the WPFF crop rotations, which was 117.3% higher on average than that of continuous cropping and 54.7% higher on average than that of other rotation treatments.

### Wheat

When compared to 2017, 2018, and 2020, wheat yields in 2019 significantly increased by 26.9, 109.2, and 84.3%, respectively (**Table 3**). The yields of wheat vary between crops and site-years. WPWF and WFWP in 2017, PWFW and FWPW in 2020 had significant yield improvements compared with 25% oil flax crop rotation sequences, with average increases of 8.5 and 27.4%, respectively. The wheat yield varied significantly between treatments in 2018. The FWPW rotation had the highest yield (2250.46 kg ha^-1^). In terms of the entire rotation cycle, the FFWP rotation sequence in 2019 had the highest wheat yield (4126.67 kg ha^-1^), with an average of 36.7, 125.3, 7.7 and 98.6% higher than that in 2017, 2018, 2019, and 2020.

### Potato

The highest yield of potato occurs in 2017, significantly increased by 29.0 and 42.0%, compared with 2018 and 2020, respectively. Crop rotation had a significant effect for potato yield in 2017 and 2019, PFWF rotation sequence observed highest yield in 2017, and FWPW recorded highest yield in 2019 (**Table 3**). The FFWP rotation series had the highest potato yield (37483.33 kg ha^-1^) in 2019.

### Effect of pre-crops on yield

During the four growth seasons, the yield of oil flax, wheat, and potato was significantly affected by year (*P*< 0.01), pre-crops (*P*< 0.01), and the interaction effects between year and pre-crops (*P*< 0.01). For the average oil flax and wheat annual yields, 2019 had a significant yield improvement compared with other years. The average yield of flax and wheat in 2019 were 134.5, 83.3, 13.8 and 25.3, 105.6, 88.3% higher than in 2017, 2018, and 2020, respectively. While the average potato annual yield was low in 2020, 2017 had a significant yield improvement compared with other years (**Table 4**). Compared with pre-crops of oil flax, wheat and potato which increased the average grain yield of oil flax, except for 2017. The pre-crops were wheat and produced the highest average oil flax yields (1367.02 and 2201.11 kg ha^-1^) in 2018 and 2019, while the pre-crops were potatoes and produced the highest average oil flax yield (2057.78 kg ha^-1^) in 2020. The highest grain yield of oil flax was achieved in 2019 with the pre-crops of wheat.

**Table 4:** Effect of pre-crops on grain yield of oil flax, wheat, and potato.

Pre-crops	Calendar year			
	2017	2018	2019	2020
	Oil flax yield			
Oil flax	845.45a	738.19c	1659.17b	1302.08c
Wheat	839.75a	1367.02a	2201.11a	1925.56b
Potato	879.11a	1175.59 b	2153.89a	2057.78a
	Wheat yield			
Oil flax	3040.07a	1836.36a	3905.93a	2162.22a
Potato	2955.92a	1816.91a	3606.67b	1826.67b
	Potato yield			
Oil flax	32966.48b	28530.93a	35775.56a	25336.67a
Wheat	42334.49a	28847.75 a	34026.67b	25584.44a
ANOVA				
Variation	P-value			
Y	**			
PC	**			
Y×PC	**			

Numbers followed by different letters within a column in a set are significantly different at P = 0.05 by the LSD test. **Significant at P ≤ 0.01.

Y, year; PC, pre-crops.

Compared with pre-crops of potato, oil flax increased the average grain yield of wheat, but only 2019 and 2020 were significantly different (**Table 4**). The grain yield of wheat significantly increased by 8.3 and 18.4% with the pre-crops of oil flax in 2019 and 2020, respectively. The pre-crops of oil flax in 2019 had the highest wheat yield (4126.67 kg ha^-1^).

Compared with wheat, the pre-crops of oil flax significantly increased potato yield in 2017, but significantly decreased potato yield in 2019 (**Table 4**). Such a phenomenon shows that the pre-crops of oil flax are beneficial to potato yields in drought years, while pre-crops of wheat are beneficial to potato yields in rainy years. In terms of the entire rotation cycle, the pre-crops of wheat in 2017 had the highest potato yield (42334.49 kg ha^-1^).

### Soil water content

**Figure 2:**
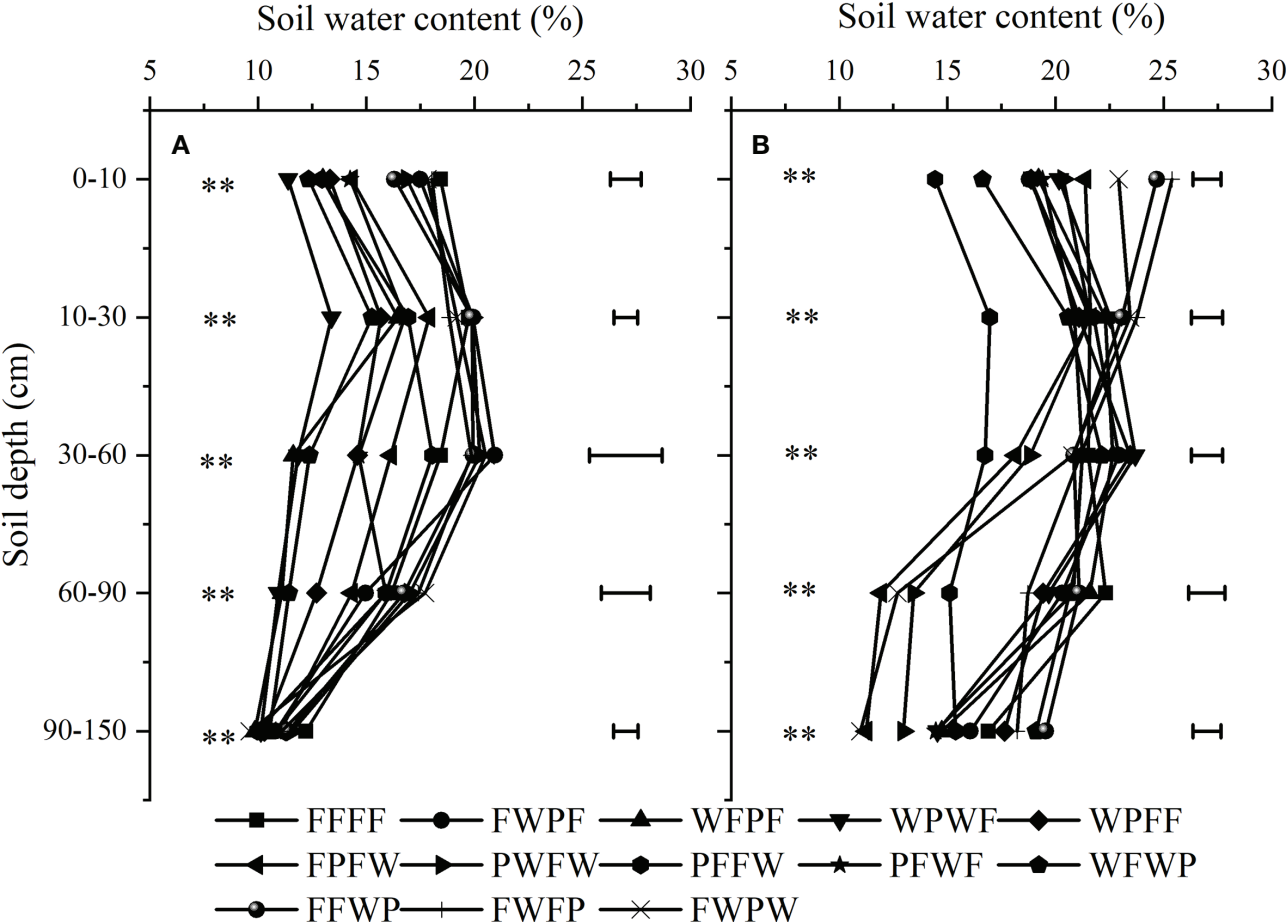
Soil water content before sowing in 2017 **(A)** and after harvest in 2020 **(B)** under different crop rotation systems. **Significant at *P* ≤ 0.01.

The results of our statistical analysis for soil water content in pre-planting and post-harvest periods indicated that soil water content in five different soil layers was significantly affected by the diversity of the crop rotation sequence (**Figure 2**). The average (0-150 cm) before sowing water content was greater under the PWFW crop rotation sequence, which had 0.7% more water content than the FFFF, and had 0.4-48.5% more water content than the other crop rotation sequences. Soil water before sowing showed that the WPWF rotation sequence provided the lowest and FFFF continuous cropping provided the highest water content in 0-30 cm soil layers. The average water content in the soil layer of the top 30 cm decreased with the increase of oil flax frequency, but this trend did not include FWPW and PWFW rotation sequences. Higher water content under the FFFF treatment could be explained by the fact that water consumption decreases due to the lower biomass of oil flax and deterioration of soil conditions. The FWPF had water contents that were 1.1% higher than the FFFF values and 0.3-48.9% greater than other crop rotation sequences in the soil layer of 30-60 cm. The water content of the FWPW soil layer was 60-90 cm, which was 9.6% higher than the FFFF and 1.8-62.2% higher than the other rotation sequences.

Significant water content variations were seen in the 0-150 cm soil layers post-harvest stage (**Figure 2**). The PFFW crop rotation sequence had a lower average water content at the post-harvest stage than the other crop rotation sequences; it contained 6.8-28.0% less water than the other crop rotation sequences and had a water content that was 22.3% less than that of FFFF. The highest average water content in FWFP rotation in the soil layer of 0-30 cm was 24.6%, the lowest in PFFW was 15.7%, and the water content of FFFF was 20.2%. Significantly higher water contents in 30-60 cm soil layers were determined in the WPWF and WPFF crop rotation sequences, which increased by 10.1 and 9.0% compared with FFFF, and increased by 3.4-41.5 and 2.3-40.1% compared with other crop rotation sequences. Significantly lower water content in soil layers (60-90 and 90-150 cm) were observed in the FPFW and FWPW crop rotation sequences compared to others. The water contents in all crop rotation sequences increased with the increasing soil depth at the post-harvest stage.

Results from our analysis showed that water content before sowing and after harvesting was affected by the diversity of crop rotation sequences that differed according to crop type and annual precipitation. Compared with bottom soil layers (30-90 cm), FFFF and FWFP practice provided higher increases in water content in the top soil layer (0-30 cm) before sowing and post-harvest, respectively.

**Figure 3:**
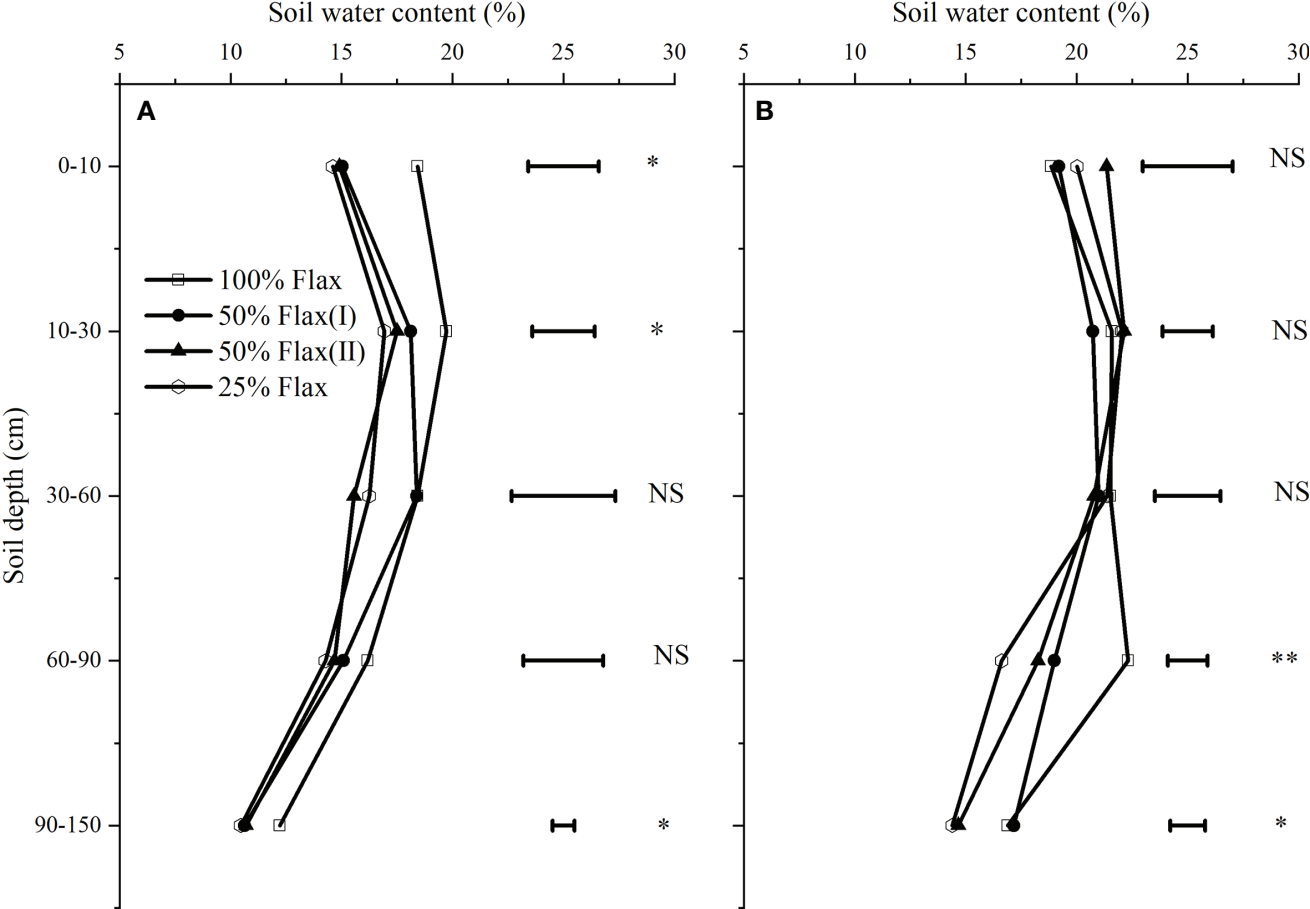
Soil water content before sowing in 2017 **(A)** and after harvest in 2020 **(B)** under different oil flax frequencies. *Significant at *P* ≤ 0.05; **Significant at *P* ≤ 0.01; NS no significant difference.

After one rotation cycle, the average water content at post-harvest increased compared with before sowing under different oil flax rotation modes (**Figure 3**). Oil flax frequency significantly affected the water content in the 0-10, 10-30, and 90-150 cm soil layers before sowing, and 60-90, and 90-150 cm soil layers after harvest, respectively. The water content of 100% oil flax at 0-10, 10-30, and 90-150 cm was 22.7, 23.7, 26.2, and 8.9, 12.8, 16.5, and 15.0, 14.1,16.7% higher than that of 50% oil flax (I), 50% oil flax (II) and 25% oil flax, respectively. The post-harvest water content was greater under the 100%Flax continuous cropping system in 60-90 soil layers than under the other oil flax frequency crop rotations. There was no significant difference between 100% oil flax and 50% oil flax (I), which significantly increased by 29.0 and 42.0%, compared with 50% oil flax (II) and 25% oil flax, respectively. According to our findings, oil flax frequency altered the water content at the sowing and harvesting stages. Deep soil water content increased as oil flax was continuously planted (60-150 cm).

### Soil water storage in fallow periods

**Figure 4:**
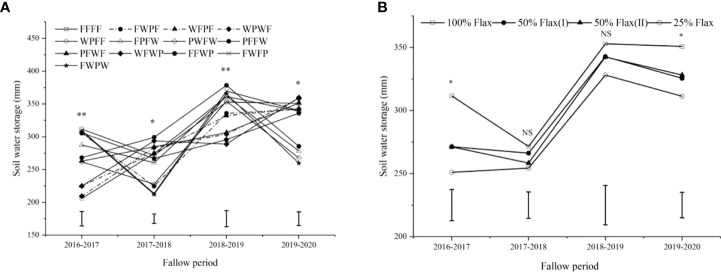
Soil water storage end of fallow period under different crop rotation systems and oil flax frequencies. *Significant at *P* ≤ 0.05; **Significant at *P* ≤ 0.01; NS no significant difference.

The results showed that SWS in fallow periods was significantly affected by the diversification of crop rotation sequences (*P*< 0.05) (**Figure 4A**). In four fallow periods, FFFF (311.57 mm), PFFW (299.08 mm), PFFW (378.36 mm), and WFWP (359.71 mm) showed the highest SWS at the end of the fallow period in 2017, 2018, 2019, and 2020, respectively. After a cycle of crop rotations, the SWS was relatively higher at the end of the fallow period, except for FPFW and FWPW. The SWS of WFPF, WPWF, and PFWF crop rotation sequences in fallow seasons decreased with the increase of rotation years.

For oil flax frequencies, there was a significant difference in SWS in 2016-2017 and 2019-2020 fallow seasons (**Figure 4B**). The highest SWS was achieved with the 100% oil flax, which was significantly higher at 14.8, 14.9, 24.1 and 7.8, 6.9, and 12.7% in 2016-2017 and 2019-2020 fallow seasons, respectively.

### Evapotranspiration and water use efficiency

During a four-year period, ETa during the oil flax growth season was mainly affected by years (*P*< 0.001), crop rotation sequences (*P*< 0.001), as well as the interaction effects of year with crop rotation sequences (*P*< 0.001), whereas ETa during the wheat and potato growth seasons was mainly affected by years (*P*< 0.001) and the interaction effects of year with crop rotation sequences (*P*< 0.01) (**Table 5**). WUE during oil flax, wheat, and potato growth seasons was mainly affected by years (*P*< 0.001), crop rotation sequences (*P*< 0.001), and the interaction effects of years with crop rotation sequences (*P*< 0.001) (**Table 5**). Compared with other years, the WUE of oil flax and potatoes increased in 2019, while the WUE of wheat increased in 2018.

**Table 5:** Effect of diversification crop rotation sequences on evapotranspiration of oil flax, wheat and potato rotation systems during 2017–2020.

Year	Flax			Wheat			Potato		
	Crop rotation	ETa (mm)	WUE (kg ha^-1^ mm^-1^)	Crop rotation	ETa (mm)	WUE (kg ha^-1^ mm^-1^)	Crop rotation	ETa (mm)	WUE (kg ha^-1^ mm^-1^)
2017	FFFF	411.94ab	2.03c	WFPF	202.28a	15.49b	PWFW	323.07a	113.43 a
2017	FWPF	396.67bc	2.17bc	WPWF	176.22a	16.22a	PFFW	345.77a	84.84 b
2017	FPFW	391.91c	1.79d	WPFF	202.94a	15.59b	PFWF	350.35a	121.04 a
2017	FFWP	412.33ab	2.59a	WFWP	193.72a	15.26b			
2017	FWFP	414.74ab	2.25b						
2017	FWPW	423.53a	2.31b						
2018	FFFF	348.74c	1.92e	FWPF	289.44ab	6.99b	WPWF	350.59a	80.98 a
2018	WFPF	356.16c	3.75a	PWFW	297.22a	6.12c	WPFF	348.47a	76.87 b
2018	PFFW	387.69b	2.94c	FWFP	286.33ab	4.32d	FPFW	339.14a	85.09 a
2018	PFWF	366.03c	3.32b	FWPW	276.87b	8.13a			
2018	WFWP	420.35a	3.33b						
2018	FFWP	357.86c	2.31d						
2019	FFFF	460.39b	3.32c	PFWF	288.79a	13.54bc	FWPF	399.91a	85.33 a
2019	WPFF	493.68ab	4.63a	WFWP	262.45b	13.24c	WFPF	401.43a	84.95 a
2019	PWFW	467.29b	4.11b	FFWP	279.48ab	14.40ab	FWPW	436.17a	85.95 a
2019	FPFW	493.05ab	4.80a	WPWF	266.99b	14.76a			
2019	PFFW	510.07a	3.54c						
2019	FWFP	491.53ab	4.40ab						
2020	FFFF	386.31a	3.18c	FPFW	235.14a	9.87ab	WFWP	396.48a	64.27 a
2020	FWPF	379.37a	5.69b	PWFW	213.70a	8.73c	FFWP	369.29a	68.39 a
2020	WFPF	378.16a	5.18b	PFFW	220.33a	10.61a	FWFP	380.51a	67.24 a
2020	WPWF	374.13a	4.95b	FWPW	192.75a	9.48b			
2020	WPFF	393.08a	3.74c						
2020	PFWF	244.41b	7.91a						
Average									
2017		408.52b	2.19b		193.79c	15.64a		339.73b	106.44 a
2018		372.81bc	2.93b		287.47a	6.39d		346.07b	80.98 b
2019		486.00a	4.13a		274.43a	13.99b		412.50a	85.41 b
2020		359.24c	5.11a		215.48b	9.67c		326.39b	66.63 b
ANOVA									
Variation		P-value							
Y		0.000(***)	0.000(***)		0.000(***)	0.000(***)		0.000(***)	0.000(***)
CR		0.000(***)	0.000(***)		0.120(NS)	0.000(***)		0.176(NS)	0.000(***)
Y × CR		0.000(***)	0.000(***)		0.004(**)	0.000(***)		0.010(**)	0.000(***)

Numbers followed by different small letters within a column in a set are significantly different at P = 0.05 by the LSD test. ***Significant at P ≤ 0.001, NS no significant difference.

FFFF, continuous oil flax; FWPF, oil flax-wheat-potato-oil flax; FPFW, oil flax-potato-oil flax-wheat; FFWP, oil flax-oil flax-wheat-potato; FWFP, oil flax-wheat-oil flax-potato; FWPW, oil flax-wheat-potato-wheat; WFPF, wheat-oil flax-potato-oil flax; PFFW, potato-oil flax-oil flax-wheat; PFWF, potato-oil flax-wheat-oil flax; WFWP, wheat-oil flax-wheat-potato; WPFF, wheat-potato-oil flax-oil flax; PWFW, potato-wheat-oil flax-wheat; WPWF, wheat-potato-wheat-oil flax. ETa, Eevapotranspiration; WUE, water use efficiency. Y, year; CR, crop rotation.

### Oil flax

The ETa of 2019 was significantly higher than in 2017, 2018, and 2020 by 19.0, 30.4, and 35.3%, respectively. The difference among crop rotation sequences was significant, and FWPW (423.53 mm), WFWP (420.35 mm), and PFFW (510.07 mm), showed better ETa through the different oil growth seasons in 2017, 2018, and 2019, respectively, while PFWF (244.41 mm) showed less ETa in 2020. The highest ETa was achieved in 2019 with the PFFW crop rotation sequence (510.07 mm), which was 20.4-30.1, 21.3-43.2, 3.3-9.2, and 29.8-108.7% higher than that of other rotation sequences in 2017, 2018, 2019, and 2020.

In four growing seasons, FFWP, WFPF, PWFW, and PFWF crop rotation sequences showed the highest WUE of 2.59, 3.75, 4.80, and 7.91 kg ha^-1^ mm^-1^ in 2017, 2018, 2019, and 2020, respectively. The PFWF crop rotation sequence in 2017 had the highest WUE (7.91 kg ha^-1^ mm^-1^), which was 256.0%, 152.7%, 84.1%, and 61.8% higher than that of other rotation sequences in 2017, 2018, 2019, and 2020 on average.

### Wheat

The crop rotation sequences only affected the WUE of wheat in 2018 and 2019, PWFW (297.22 mm) and PFWF (288.79 mm) displayed relatively high ETa. The highest ETa of wheat was achieved with the PWFW in 2018, which was higher than the 53.4, 8.3, and 37.9 (average) that in 2017, 2019, and 2020, respectively.

In the four growing seasons, the average WUE of wheat ranked in the following order: 2017 > 2019 > 2020 > 2018 (**Table 5**). For crop rotation sequences, WPWF (2017), FWPW (2018), FFWP (2019) and PFFW (2020) had significantly higher WUE than other treatments, and they increased by 4.0-6.3, 16.3-88.2, 2.5-11.5, and 7.5-21.5%, respectively. In terms of the entire rotation cycle, the WPWF rotation sequence in 2017 had the highest WUE of wheat (16.22 kg ha^-1^ mm^-1^), with an average of 3.7, 153.8, 16.0 and 67.7% higher than that in 2017, 2018, 2019 and 2020.

### Potato

Compared with other years, ETa in 2019 increased significantly by 21.42%, 19.20, and 26.38%, respectively. For crop rotation sequences, the difference among crop rotation sequences was not significant.

For the years, 2017 had significantly higher WUE than 2018, 2019 and 2020, and they increased by 31.4, 24.6, and 59.8%, respectively. The crop rotation sequences only affected the WUE of potatoes in 2017 and 2018, PFFW (84.84 mm) and WPFF (76.87 mm) showed a relatively low ETa. The WUE of potato under the PFWF rotation sequence in 2017 was the highest, which was 49.5, 41.7, and 81.7% higher than that in 2018, 2019 and 2020, respectively.

**Figure 5:**
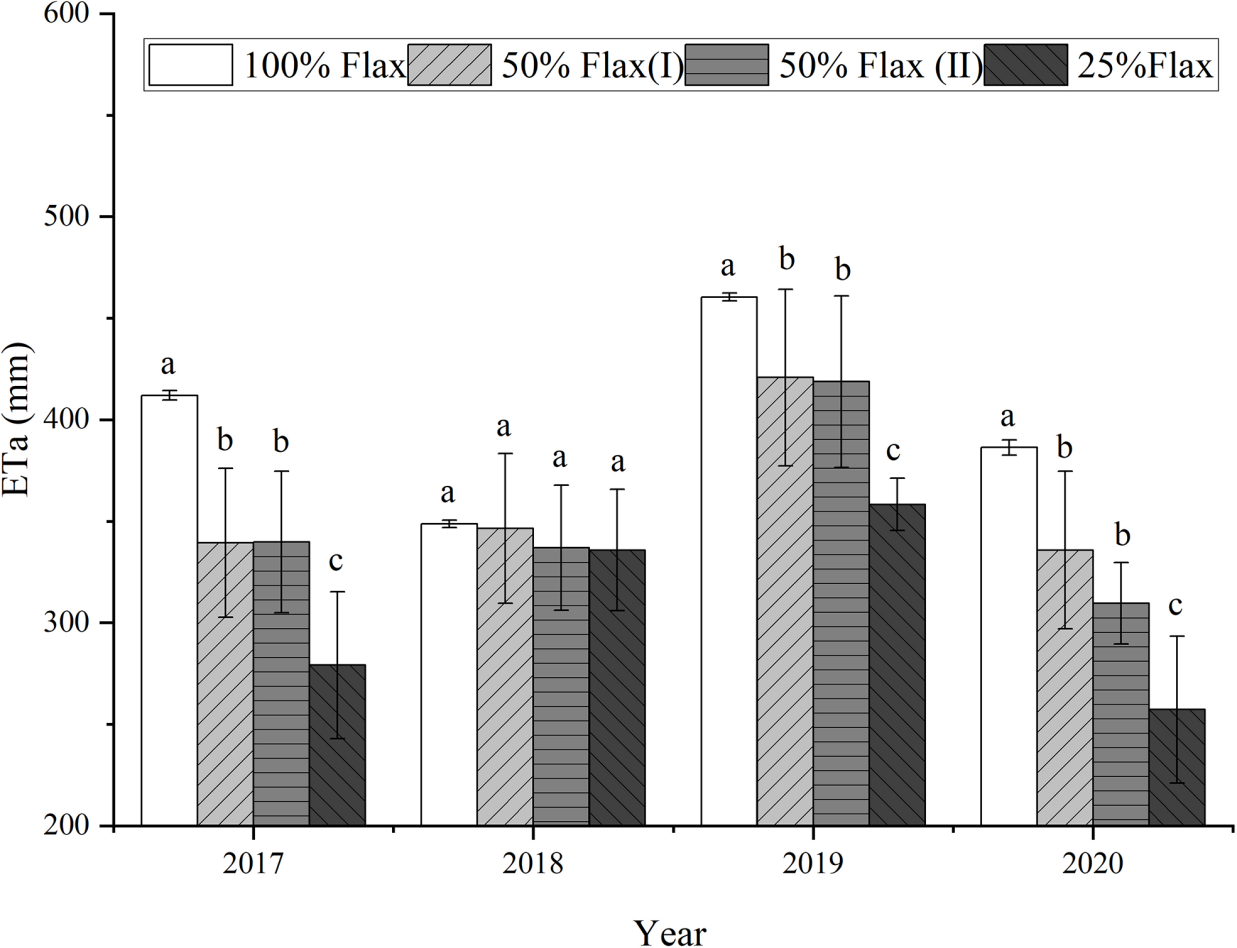
The average evapotranspiration of each oil flax frequencies cropping system for each cropping year from 2017 to 2020. Different small letters show significant difference according to LSD test at *P*< 0.05 level.

In 2018, the difference in ETa among oil flax frequencies was not significant. The ETa varied with oil flax frequencies, being the maximum with 100% oil flax, less with 50% oil flax, and the minimum with 25% oil flax in 2017, 2019 and 2020 (**Figure 5**). The difference in ETa was not significant between 50% Oil flax (I) and 50% Oil flax (II) in four years. 100% oil flax showed the highest ETa of 460.39 mm in 2019, and 25% oil flax showed the lowest ETa of 254.73 mm in 2020.

### Effects of oil flax frequency in a rotation on RCV of grain yield and WUE

**Figure 6:**
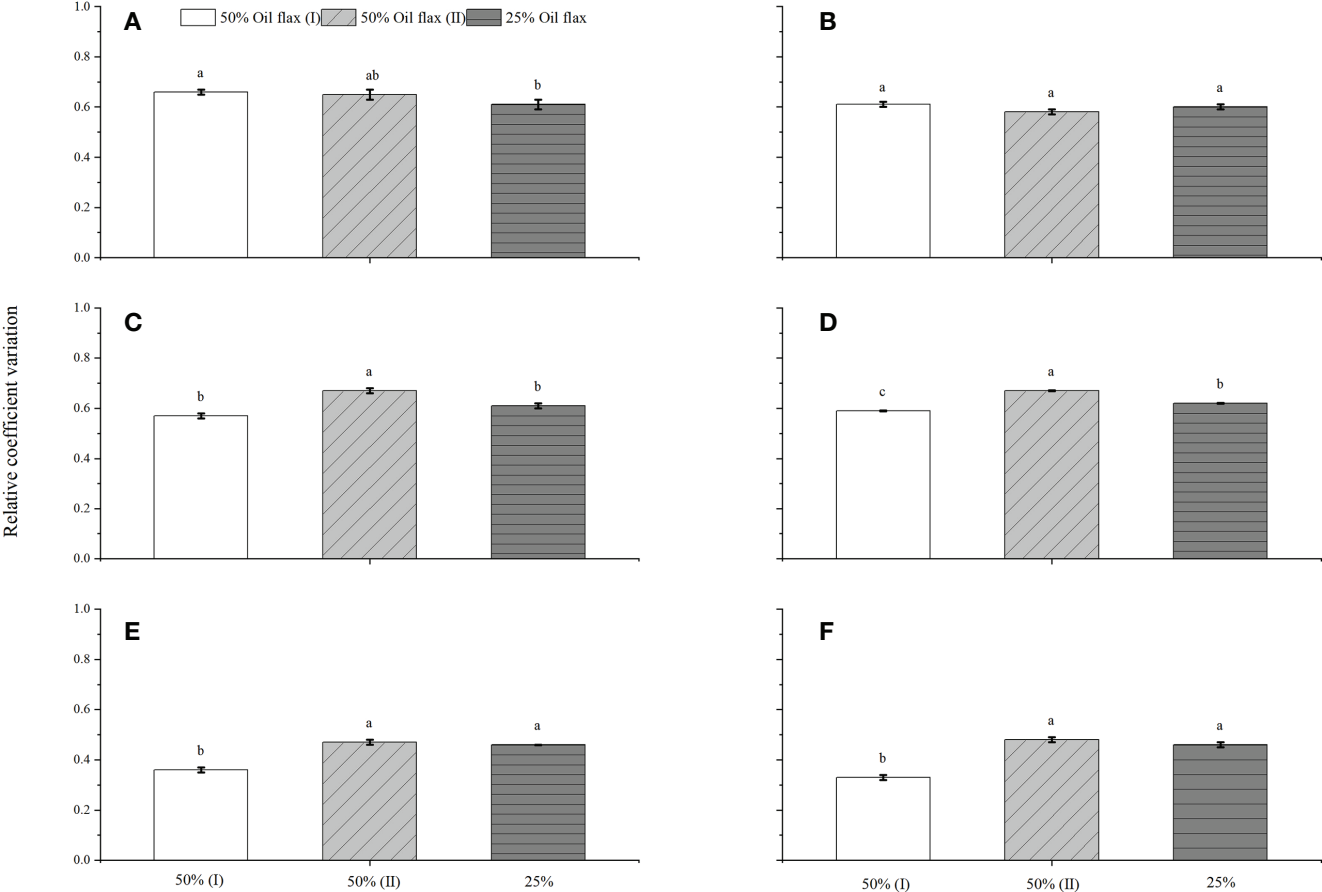
Effects of frequency of oil flax in a rotation cycle on relative variation coefficient of yield of oil flax **(A)**, wheat **(B)**, and potato **(C)**, relative variation coefficient of WUE of oil flax **(D)**, wheat **(E)**, and potato **(F)**. The bar with different letters means the significant difference at 0.05 levels. Different small letters show significant difference according to LSD test at *P*< 0.05 level.

For oil flax, the 25% oil flax significantly decreased the RCV of grain yield by 7.6%, compared with 50%(I) oil flax (**Figure 6A**), while the RCV of WUE was not significantly different in oil flax frequency (**Figure 6B**). For wheat, 50% oil flax (I) decreased RCV of grain yield and WUE by 17.5 and 13.9%, respectively (**Figures 6C, D** **)**, compared with 50% oil flax (II). Like wheat, 50% oil flax (I) decreased potato RCV of yield and WUE by 30.6 and 48.8%, respectively (**Figures 6E, F** **)**, compared with 50% oil flax (II).

## Discussion

Compared with continuous monoculture systems, well-planned crop rotations can be improve crop yields and the sustainability of production systems by promoting efficient nutrient cycling, efficient use of natural resources (especially water), maintaining long-term land productivity, and controlling pests and diseases, thereby reducing reliance on external inputs (Walter and Andrea, 2011). A diversity crop rotation system, especially in dry-land is more sustainable if supported by improved water productivity rather than solely on crop yield (Schlegel et al., 2019).

Beneficial crop management practices in arid and semi-arid regions, such as crop rotation, especially well-planned diversity crop rotations, not only increase the availability of, or maximize the efficient use of, limited resources such as water and soil nutrients for use by crops, but also increased crops yield and improved the profit of the producer (Schlegel et al., 2017). Compared with continuous monoculture systems, crop rotation has long been recognized as a beneficial practice that enhances SWS capacity (Walter and Andrea, 2011; Bowles et al., 2020), especially for diverse crop rotation systems (Schlegel et al., 2017). In the current study, PWFW before sowing in 2017 and the FFWP crop sequence after harvest in 2020 had higher SWC, compared with continuous cropping of oil flax, and the current season crops were all potatoes. After a 4-year crop rotation cycle, the water content after harvest was higher than that before sowing, which was 28.5% higher than that before sowing on average. This may be because the annual rainfall in the test area was mainly concentrated in July to September, and this mismatch phenomenon between rainfall and crop growth resulted in higher water content after harvest than before sowing. The continuous cropping of oil flax in the four growing seasons also showed higher SWC, which was related to evapotranspiration. The continuous cropping of oil flax had a lower grain yield (**Table 3**), less water consumption, and lower evapotranspiration (**Table 5**), resulting in a higher SWC.

The loss of large amounts of soil moisture to unproductive purposes has been considered as a main factor resulting in land degradation in the Loess Plateau (Duan et al., 2020). Because of the uneven distribution of precipitation in this region, water resources are sometimes insufficient to sustain normal crop growth and even lead to surface runoff (Bu et al., 2013; Chen et al., 2015). The SWS at sowing and effective precipitation during the crop growth season are the main factors that determined crop yield (Sang et al., 2016). This indicates that soil moisture at sowing time plays a key role in crop production in dry-land agriculture, especially in drought years. In this study, there was a long-term winter fallow period (from late July after the wheat harvest to early April before oil flax sowing the following year; from late August after the oil flax harvest to early April before oil flax sowing/late March before wheat sowing/early May before potato sowing the following year; from late October after the potato harvest to early April before oil flax sowing/late March before wheat sowing the following year) followed by the wheat, oil flax, or potato growth season. Thus, better soil water conservation in the fallow period would provide a better soil water condition for the subsequent production of oil flax, wheat, or potatoes.

Fallow is defined as the time period between crop harvest and following crop planting (Schlegel et al., 2017), which is widely used in rain-fed agriculture to maximize SWS, make sure the subsequent crops are growing properly and thus decrease the risk of crop failure (Pala et al., 2007). Conventional grain production systems in the Loess Plateau have relied on winter fallow to accrue additional water to stabilize crop yields. The fallow period lengthens the time between crops (pre-crops and subsequent crops) so more precipitation can be absorbed and stored in the soil for subsequent crop production (Nielsen and Vigil, 2017). Greater fallow soil water accumulation results in differences in soil water at planting between diverse crop rotation systems (Gan et al., 2016). Soil water content before sowing were affected by many factors, such as precipitation in the fallow period, pre-crops type and management, weather conditions during the growing season, land cover and so on (Schlegel et al., 2019). So, the SWS at the end of fallow or before sowing plays a key role in the performance of upland crops (Schlegel et al., 2017). Meanwhile, Sainju et al. (2021) reported that reduced crop yield in the previous year likely increased pre-plant soil water with continuous wheat, and diversified crop rotations had greater pre-plant soil water content than that continuous mono-cropping (Lenssen et al., 2014; Lenssen et al., 2018). In addition, the pre-crops also had a great influence on the SWS in the fallow season.

In the current study, except for continuous cropping of oil flax, the SWS in the fallow season and the pre-crops synergies affected the grain yield of oil flax. The higher SWS of FFWP, FWFP, and FWPW rotation sequences in 2017, WFWP in 2018, and the pre-crops were WP or PW in 2019 and 2020, obtained a higher grain yield of oil flax (**Table 3**). In conclusion, higher SWS in the fallow season, or when the pre-crops were WP or PW, could obtain a higher grain yield of oil flax. The grain yield of wheat also showed a similar pattern. The highest SWS of WFPF rotation sequences in 2017 and PFFW in 2020, and the pre-crops was WF in 2018, which obtained a higher grain yield of wheat (**Table 3**). But the trends were different in 2019, such as Nielsen and Vigil (2017) reported that the longer-term effects of pre-crops on subsequent soil water use would be difficult to predict in dry farming areas with variable precipitation. It follows that crop sequence in a crop rotation system has a great influence on SWS in the fallow season. This may be because a crop grown in year 1 of a rotation could have an impact on the water use of subsequent crops in years 2, 3, and 4 of the rotation, especially when rainfall is below average for the region (Kirkegaard and Ryan, 2014).

Crop frequency in the sequence of the crop rotation system affect the output by affecting soil nutrients and water, which is finally shown to have a significant effect on average annual net revenue (Brennan and Smith, 2017; Khakbazan et al., 2020). Our results suggest that oil flax frequency in the rotation cycle significantly affected soil water content before sowing and after harvest in the next rotation cycle (**Figure 2**). Water content was significantly higher in soil layers (0-10, 10-30, and 90-150 cm) in the 100% oil flax practice at the pre-plant stage and in soil layers (60-90 cm) in the post-harvest practice compared to the 50% oil flax and 25% oil flax practices, and water content decreased with decreasing frequency of oil flax. This may be related to the water consumption of the crop itself; the water consumption of oil flax was lower than that of wheat and potatoes, and maintaining a higher soil moisture content before planting in the next rotation cycle (Zhao et al., 2020a). Long-term continuous cropping of oil flax resulted in a decrease in the number of plants and an increase in evapotranspiration, which resulted in a decrease in soil water content at post-harvest. Furthermore, oil flax frequency significantly affected soil water storage in the 2016-2017 and 2019-2020 fallow seasons, but there was no significant difference in soil water storage in the 2017-2018 and 2018-2019 fallow seasons. However, 100% oil flax frequency in the four fallow seasons showed the highest soil water storage, which was 311.57, 271.49, 352.91, and 350.83 mm, respectively. It follows then that both continuous cropping years and crop frequency could affect soil water, which is also confirmed by Li et al., 2021b on maize and Khakbazan et al. (2020) on pulse crops.

Diversification crop rotation had a greater effect on annualized grain yield, water use, and productivity (Sainju et al., 2021). The use of diversification rotations has been largely associated with beneficial effects on crop yield (Álvaro-Fuentes et al., 2009). Diversification crop rotation influences crop yield through its effects on soil nutrients and carbon, soil physical and chemical properties, weed and pest cycles, soil water availability, and soil microbial community structure, etc (Attia et al., 2015). The analyzed results showed that diversification crop rotation increased the grain yield of oil flax, and the grain yield was highest when the crop rotation sequence was WPF or PWF. This may be related to the higher SWS accumulated when the pre-crops were WP or PW in the winter fallow season. The FFFF had the lower grain yield in all 4 growth seasons. This could be explained by continuous cropping obstacles and deteriorating soil conditions. The result is consistent with previous findings (Wang et al., 2020). The grain yield of continuous oil flax in 2018 decreased by 27.85% compared with that in 2017, and in 2020 it decreased by 25.37% compared with that in 2019. It is related to the residual water in the soil because there was more rainfall in the current season, which means crops can use more residual water in the next season, and the grain yield was relatively higher (Schlegel et al., 2019). The precipitation in 2018 was 360.9 mm, which was much higher than that in 2017, and resulted in a much higher grain yield in 2019 (**Figure 1**). In addition, the grain yield of FFFF decreased with the increasing number of years. That’s because crops under continuous cropping can be adversely affected by allelopathic or autotoxic effects Li et al., 2021c.

Soil nutrients absorbed by the previous crop would affect the soil quality and eventually be reflected in the growth and development of the subsequent crop (Zhang et al., 2010). In this study, all wheat-containing rotations showed the highest grain yield of wheat in the FFW rotation sequence except for 2018. The effect of rotation on crop yield also depends on the type of pre-crops, ranging from 2% for Triticeae pre-crops to 27% for grain leguminous pre-crops (Zhao et al., 2020b; Fang et al., 2021). In the current study, the pre-crops had no significant difference in the yield of flax in 2017, wheat in 2017 and 2018, and potatoes in 2018 and 2020 (**Table 3**). Compared with oil flax pre-crops, the grain yield of oil flax under wheat and potato pre-crops was significantly increased by 85.4, 32.7, 47.9, and 59.3, 29.8, and 58.0% in 2018, 2019 and 2020, respectively. Also, inter-annual rainfall is another key factor affecting crop yield in crop rotation systems (Zhao et al., 2020b). Niu et al. (2017) reported that all phases of plant development were influenced by rainfall during the crop growing period, which plays a more important role in crop yield than pre-planting residual soil water. In the rotation cycle we studied, 2019 saw more rain, so the grain yield of oil flax and wheat was higher than that in other years. Moreover, the highest grain yields of oil flax and wheat were achieved when the pre-crops were WP or PW, and FF.

In arid and semi-arid regions, crop management practices that reduce evaporation and increase SWS can improve crop yields, and the reduction of soil water loss by evaporation is important for the protection of SWS (Hulugalle et al., 2017). Previous studies have shown that diversified crop rotations have a significant effect on Eta and may affect crop water productivity (Zhang et al., 2019b). In the current study, crop ETa varied with crop rotations and pre-crops in various years. In 2017 and 2020, the average ETa of oil flax under all crop rotation sequences was lower than that under FFFF and higher than that under FFFF in 2018 and 2019. This demonstrated that the crop rotation sequence and inter-annual rainfall synergistically affect ETa. The highest ETa of oil flax appeared in the PWF crop rotation sequence, in 2017 and 2018, while it appeared in the PFF crop rotation sequence in 2019 and 2020. The crop rotation sequence only affected the ETa of wheat in 2018 and 2019, and the highest ETa was achieved with the rotation sequence of PWFW and PFWF. Consequently, arranging the sequence of crops in diversified crop rotations needs to consider the water requirements of different crops; in this way, high water requirement crops should be arranged after low water requirement crops (Garbelini et al., 2022).

System-wide water saving can be achieved by reducing unproductive purpose evaporation. Diversified crop rotations are an effective measure to reduce evaporation and increase crop WUE (Yang et al., 2015). Compared with continuous cropping, diversified crop rotations can enhance precipitation and water utilization efficiency (Johnston et al., 2002; Lenssen et al., 2014). In the current study, the average WUE of oil flax under all crop rotation sequences was higher than that of continuous oil flax in all 4 growth seasons. In agreement with a previous study on the Loess Plateau, the rotations gave significantly improved WUE compared to the monoculture (Huang et al., 2003). In the long-term crop rotation experiments, rotations had significant effects on crop production, and thus on WUE. A small gain in the balance between water transpired by crops and water lost by evaporation can significantly increase crop productivity and WUE (Pala et al., 2007). The WUE was different between the rotation phases, growing seasons, and the interaction of both factors in this study. As a result, the highest WUE of oil flax, wheat and potato were achieved for PFWF (7.91 kg ha^-1^ mm^-1^), WPWF (16.22 kg ha^-1^ mm^-1^) and PFWF (121.04 kg ha^-1^ mm^-1^) crop sequences in 2020, 2017, and 2017, respectively. There is a phenomenon we found that the maximum grain yield and WUE of oil flax and wheat were not consistent, which indicates that the evaluation of the productivity of a crop rotation system needs to take into account many factors (such as weeds, pest, and disease infections, net return, etc.), and it is still insufficient to consider only the yield and WUE.

In summary, continuous oil flax significantly reduced the WUE of oil flax compared with crop rotation, and the WUE of oil flax, wheat, and potato had different responses to the crop rotation sequences and the pre-crops in different years. The WUE of oil flax was the most affected by the rotation sequence and pre-crops, followed by wheat, and the lowest in potato. This may be related to the crop ETa and the occurrence frequency of the three crops in a rotation cycle. The ETa of oil flax was higher than that of wheat and close to that of potatoes (**Table 5**), while potatoes were grown for only one season during the entire rotation cycle.

The RCV reflects the variation in grain yield and WUE among the experimental years, with a lower RCV indicating higher stability of the agronomic production system (Zhang et al., 2020). In our study, 25% oil flax decreased the RCV of oil flax grain yield, while there was no significant difference in the RCV of WUE among different flax frequencies. The 50% oil flax (I) decreased the RCV of wheat and potato economic yield and WUE, respectively. This suggested that 25% oil flax stably increased oil flax, and 50% oil flax (I) stably increased wheat and potato productivity in this semiarid rain-fed area, decreasing the risk of biotic stresses to crop productivity.

## Conclusions

Crop rotation is an effective way of increasing crop yield and making fuller use of arable land. In the 4-year-cycle field experiment, the crop rotation sequences of FFWP, FWFP, FWPW and 100% oil flax, and 50% oil flax (II) showed better soil water conditions in the pre-planting and post-harvesting periods. Soil water storage varies by year and rotation sequences during fallow periods. The average ETa of oil flax was the highest, followed by potato, and the lowest of wheat, and continuous oil flax contributed to a higher ETa. Diversity crop rotations significantly increased the grain yield and WUE of oil flax, compared with continuous oil flax, and the grain yield was highest when the crop rotation sequences were WPF or PWF, while the WFPF crop rotation sequence had the higher WUE of oil flax. Compared with potato pre-crops, oil flax increased the grain yield of wheat, especially when the crop rotation sequence was FFW, while the WPWF crop rotation sequence had the higher WUE of wheat. There was no clear effect of the pre-crops on the yield and WUE of potatoes. It can be seen that the treatments with maximum grain yield and WUE of oil flax and wheat were not consistent. 25% oil flax frequency had the higher stability of the oil flax, and 50% oil Flax (I) frequency had the higher stability of the wheat and potato production systems. Thus, the wheat-potato-oil flax or potato-wheat-oil flax rotation can increase oil flax grain yields, and wheat-oil flax-potato-oil flax markedly improved oil flax WUE. Oil flax-oil flax-wheat rotation can increase wheat grain yields and wheat-potato-wheat-oil flax markedly improved wheat WUE.

## Data availability statement

The raw data supporting the conclusions of this article will be made available by the authors, without undue reservation.

## Author contributions

Author ZC analyzed the data and prepared the first and draft. BY as a project administration. YG conceived the conceptualization and methodology for the experiments. BW, YFW, and YX investigated the manuscript. PX, HW, WM, YZW, and XM helped in experiments and data collection. All authors contributed to the article and approved the submitted version.

## Funding

This work was supported by the Research Program Sponsored by Gansu Provincial Key Laboratory of Aridland Crop Science, Gansu Agricultural University (GSCS-2020-Z6), the National Natural Science Foundation of China (31760363, 32260551), the China Agriculture Research System of Construct Special (CARS-14-1-16), Gansu Education Science and Technology Innovation Industry Support program (2021CYZC-38), the Fuxi Outstanding Talent Cultivation Plan of Gansu Agriculture University (Gaufx-02J05); the Education Science and Technology Innovation Project of Gansu Province (2021CXZX-366).

## Acknowledgments

Authors are thanks to editors and reviewers for providing valuable comments for improving manuscript.

## Conflict of interest

The authors declare that the research was conducted in the absence of any commercial or financial relationships that could be construed as a potential conflict of interest.

## Publisher’s note

All claims expressed in this article are solely those of the authors and do not necessarily represent those of their affiliated organizations, or those of the publisher, the editors and the reviewers. Any product that may be evaluated in this article, or claim that may be made by its manufacturer, is not guaranteed or endorsed by the publisher.
